# Influence of antibiotic pressure on multi-drug resistant *Klebsiella pneumoniae* colonisation in critically ill patients

**DOI:** 10.1186/s13756-019-0484-8

**Published:** 2019-02-14

**Authors:** Jesus Ruiz, Monica Gordon, Esther Villarreal, Juan Frasquet, María Ángeles Sánchez, María Martín, Álvaro Castellanos, Paula Ramirez

**Affiliations:** 1 Intensive Care Unit, IIS La FE, Hospital Universitario y Politécnico La Fe, Valencia, Spain; 2 Microbiology Department, Hospital Universitario y Politécnico La Fe, Valencia, Spain; 3 Intensive Care Unit, Hospital Universitario y Politécnico La Fe, Valencia, Spain

**Keywords:** K pneumoniae, Antibiotic, Critcal care, Multidrug resistance, Colonization

## Abstract

**Background:**

The aim of this study is to evaluate the risk factors for colonisation by multidrug resistant (MDR) *K. pneumoniae* in a critical care unit and the relationship between colonisation and the antibiotic pressure exerted by the antimicrobial treatments received by patients.

**Methods:**

A prospective observational was designed. Patients admitted for more than 48 h to an intensive care unit were included. Samples for surveillance cultures were obtained from all the patients upon admission and once a week. The association between risk factors and colonisation by MDR *K. pneumoniae* was determined by logistic regression. A Cox regression model was used to evaluate the effect of the use of antimicrobials on the colonisation rate. An ARMIA model was used to investigate the association between the incidence of colonisation by MDR strains and the global consumption of antimicrobials in the unit.

**Results:**

One thousand seven hundred twenty-five patients were included, from which 308 (17.9%) were positive for MDR *K. pneumoniae*. In the multivariate analysis, hospitalisation for longer than 7 days together with respiratory infection and administration of any antibiotic was associated with increased MR *K. pneumoniae* colonisation. Patients who received antibiotics for more than 48 h were colonised earlier than patients who did not receive antibiotic treatment [HR: 2.16 (95%CI:1.55–3.03)]. The ARIMA model found a significant association between the monthly colonisation rate for MR *K. pneumoniae* and the consumption of cephalosporins and carbapenems in the previous month.

**Conclusion:**

Individual antibiotic administration and the global antibiotic pressure of cephalosporins and carbapenems are associated to an increased colonisation by MDR *K. pneumoniae* strains.

## Background

*Klebsiella pneumoniae* is now one of the microorganisms most commonly associated with healthcare and nosocomial care-associated infections, mainly in patients admitted to critical care units [1–3]. From the first time *Klebsiella* spp. isolates were described as producing extended-spectrum b-lactamases (ESBL) in 1983, a progressive increase in multiresistant (MR) strains of this species has been observed worldwide [4]. The increase in the number of hospital infections caused by MR strains of *K. pneumoniae* is especially worrisome, as more than 30% of episodes of nosocomial bacteraemia caused by this species are now MR strains, reaching around 45% of episodes in critically ill patient [5]. The demonstrated association between resistance and morbi-mortality, hospital stay length, and health costs [6, 7], as well as the limited therapeutic options available, highlights the importance of controlling and minimising the expansion of this species.

The description of the risk factors associated with infection by MR strains of species such as *Pseudomonas aeruginosa* or *Acinetobacter baumannii* in critical patients has been the subject of multiple studies, with previous exposure to various different antibiotics being one of its highest risk factors [8–11]. However, there is little data available about the effect of antimicrobial consumption on colonisation by MR *K. pneumoniae*, even though this phenomenon is of special interest because, in most cases, these have been used prior to infection [12, 13]. Thus, the objective of this work was to evaluate the risk factors for colonisation by MR strains of *K. pneumoniae* in a critical care unit and to analyse the relationship between this colonisation and the antibiotic pressure exerted by the antimicrobial treatments received by patients, as well as by the overall consumption of antibiotics in the unit.

## Methods

We designed a prospective and observational study which included adult patients admitted for more than 48 h to an intensive care unit (ICU) in a tertiary hospital between January 2014 and December 2016. The ICU where the study was conducted attends patients with medical pathologies; it has 24 beds and in recent years has had an average of 1592 admissions per year. Despite the introduction of various measures, since 2013 the unit has presented an endemic situation of *K. pneumoniae* colonisation, with a colonisation pressure (ratio of patients colonised with MR *K. pneumoniae*/total number of patients per day) of 14.8% this year. Patients admitted for less than 48 h and with colonisation known prior to admission to the unit, or who were colonised by MR species other than *K. pneumoniae* during their hospitalisation, were excluded from the study.

Following the internal operating protocols of our ICU, samples from all the patients in the unit were submitted for surveillance cultures upon admission and once a week thereon, to screen for MR bacteria colonisation. The samples consisted of a rectal swab, an oropharyngeal swab, and a bronchial aspirate from intubated patients. In addition, where a case of clinical infection was suspected, samples were obtained for microbiological study.

The clinical samples were processed according to our usual methodology. The cultures were incubated at 35 °C for 48 h, followed by a subculture on selective chromogenic medium for ESBL-producing enterobacteria and carbapenemases. The antibiogram of the colonies obtained from these cultures was identified and studied according to standardised methods used in the microbiology laboratory. Strains of *K. pneumoniae* were classified as MR following the criteria established by the European Centre for Disease Prevention and Control (ECDC) [14], considering MR strains to be those which were resistant to three or more groups of antimicrobials and carbapenemase-producing strains those isolates presenting either minimum inhibitory concentration (MIC) of 0.125 mg/liter to meropenem and/or ertapenem and/or 1 mg/liter to imipenem [15].

Data about the antibiotic exposure were collected during the follow-up period, both individually from the patients included in the study cohort, as well as globally in the unit. The overall consumption of antibiotics in the unit was calculated based on the criteria established by the WHO, by calculating the daily defined dose (DDD) per 100 patients per day [16]. For each of the patients included in the study, we recorded the antibacterial treatment received during their stay in the ICU, including any digestive-tract decontaminating solutions administered, until colonisation by *K. pneumoniae* was detected or the patient was discharged from the unit.

### Statistical analysis

Statistical analysis was performed with the Stata (v.12.0) program. For the comparison of variables between patients with and without colonisation, chi-square or Fisher’s exact tests were used for categorical variables and Student t-tests were implemented for continuous variables. The association between risk factors and colonisation by strains of MR *K. pneumoniae* was determined by logistic regression; any variables with a probability (p) value of less than 0.2 in the univariate model were included in the multivariate model. A Cox regression model was used to evaluate the effect of the use of antimicrobials for more than 48 h on the colonisation rate. Furthermore, we used an autoregressive integrated moving average (ARIMA) model with monthly time periods to investigate the association between the incidence of colonisation by MR strains and the global consumption (DDD/100 stays) of antimicrobials in the unit during the same month and the previous month. Any tests with a *p* < 0.05 value were considered significant.

## Results

During the study period there were a total of 1994 ICU admissions whose stay exceeded 48 h and from whom an epidemiological surveillance sample was collected. Of these, 269 (13.5%) were excluded because they were colonised by other MR species during their stay in the unit. These species were: *Enterobacter cloacae* in 108 (5.4%) patients, *P. aeruginosa* in 68 (3.4%), Methicillin-resistant *S. aureus* (MRSA) in 48 (2.4%), *A. baumannii* in 32 (1.6%), and other species in 13 (0.7%) patients. A total of 1725 patients were included, from which a total 9042 surveillance samples were obtained; 1256 samples (13.9%), corresponding to 308 (17.9%) patients, were positive for MR *K. pneumoniae*, and of these, 536 (from 119 patients) were ESBL-producing strains, 745 (183 patients) were carbapenemase-producing strains, and 25 samples (6 patients) contained both strains. Of the total number of positive samples, 826 (65.8%) were from rectal swabs, 291 (23.2%) from pharyngeal swabs, and 139 (11.0%) from bronchial aspirates.

**Fig. 1:**
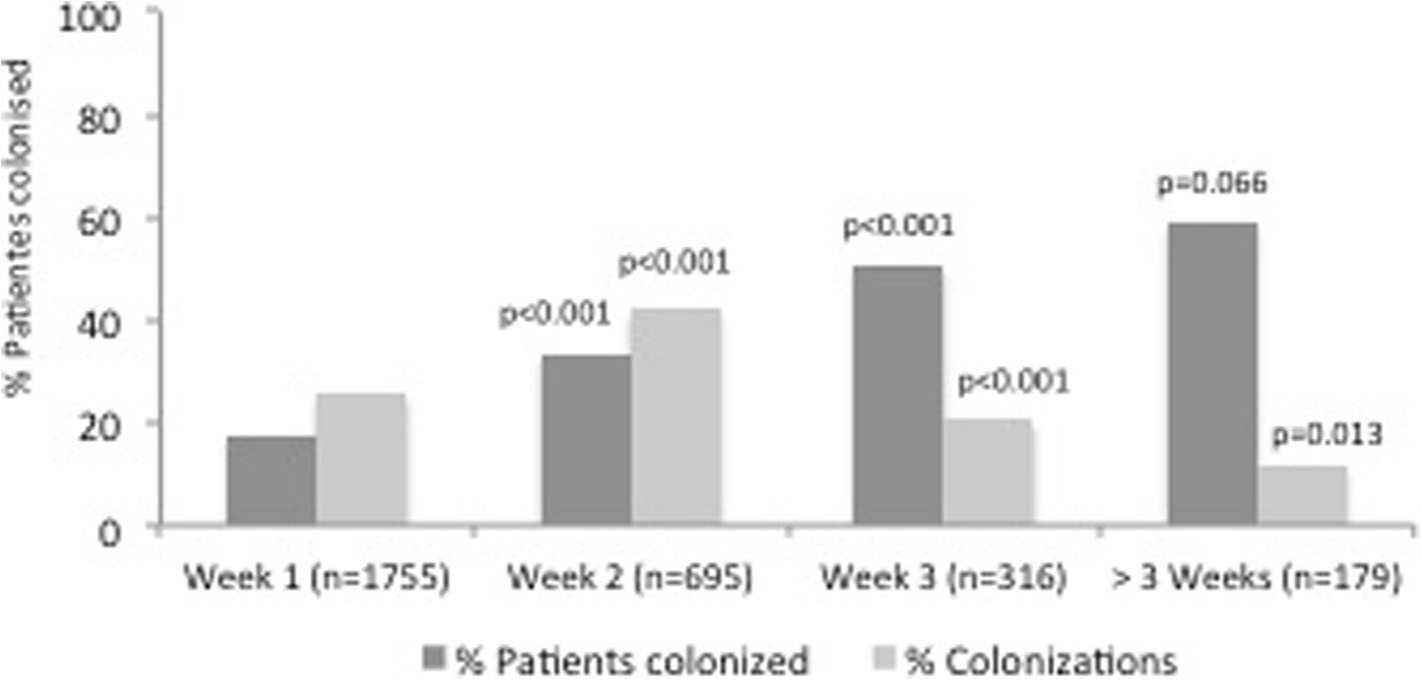
Percentage of patients colonised in the unit and new colonisations according to the number of weeks of stay in the unit. *P*-values represent the differences in percentages of patients colonised and new colonisations between each week and the previous one

The demographic and clinical characteristics of these patients are shown in Table 1. The percentage of patients colonised, according to the number of weeks they stayed in the unit, as well as the percentage of new colonisations found for each week of stay, are shown in Fig. 1; most cases were identified after the second week of hospitalisation in the unit. The colonised patients had a longer mean stay length [19.3 ± 16.1 days vs. 7.8 ± 7.3 days); *p* < 0.001] and a higher mortality rate (30.8% vs. 17.1%; *p* < 0.001) in the unit compared to the non-colonised patients (Table 2).

**Table 1:** Characteristics of patients included in the study

	Total	Patients colonized by *K. p. Pneumoniae*	Patients not colonized by *K. pPneumoniae*	p
	*n* = 1725	*n* = 308	*n* = 1417	
Age (SD)	60.9 (15.2)	58.1 (14.9)	61.5 (15.2)	< 0.001
Male (%)	1097 (63.6)	203 (65.9)	895 (63.2)	0.364
Length of stay (SD)	9.85 (10.46)	19.3 (16.1)	7.8 (7.3)	< 0.001
^1^ICU mMortality (%)	338 (19.6)	95 (30.8)	243 (17.1)	< 0.001
APACHE II (median, IQR)	19 (14–24)	21 (18–25)	18 (13–24)	< 0.001
Hematologic disease	120 (6.9)	19 (6.2)	101 (7.1)	0.531
Solid organ tTransplantation	73 (4.2)	18 (5.8)	55 (3.9)	0.133
Immunosuppression	267 (15.5)	60 (19.4)	207 (14.6)	0.035
Diabetes	351 (12.4)	65 (21.1)	268 (20.2)	0.722
Cirrhosis	64 (3.7)	18 (5.8)	46 (3.2)	0.028
^2^EPOCCOPD	351 (20.3)	93 (30.2)	258 (18.3)	< 0.001
Mechanical vVentilation (%)	408 (23.7)	85 (27.6)	324 (22.8)	0.077
Renal rReplacement therapy (%)	142 (8.2)	21 (6.8)	121 (8.5)	0.319
Parenteral nNutrition	753 (34.1)	103 (33.4)	408 (28.8)	0.109
Origin				0.179
Emergency dDepartment	793 (46.0)	126 (40.9)	667 (47.1)	
Hospitalization	378 (21.9)	92 (29.9)	461 (32.5)	
Other hHospital	534 (30.9)	90 (29.2)	282 (19.9)	
Cause of admission				< 0.001
Respiratory infection	321 (18.6)	77 (25.0)	243 (17.1)	
Sepsis/Septic sShock	181 (10.5)	24 (7.8)	156 (11.0)	
Cardiovascular disease	567 (32.9)	60 (19.5)	507 (35.8)	
Other	976 (56.7)	147 (47.7)	511 (36.1)	

^1^ICU: Intensive care unit; ^2^COPD: Chronic obstructive pulmonary disease

**Table 2:** Results from the univariante analysis

	OR (IC95%)	P
Age > 60 years	0.86 (0.40–1.83)	0.700
Length of stay > 7 days	6.52 (4.84–8.52)	< 0.001
APACHE II > 20	1.98 (0.89–2.62)	0.092
Hematologic disease	0.84 (0.17–3.97)	0.825
Solid Organ Transplantation	1.59 (0.31–8.26)	0.580
Origin	1.38 (1.21–1.57)	0.168
Immunosuppression	1.27 (0.47–3.40)	0.641
Diabetes	0.63 (0.26–1.56)	0.320
Cirrhosis	1.92 (0.36–10.37)	0.477
^1^COPD	0.89 (0.82–1.74)	0.380
Mechanical ventilation	1.34 (0.61–2.95)	0.465
Renal replacement therapy	1.66 (0.91–2.14)	0.134
Parenteral Nutrition	2.18 (1.01–4.69)	0.045
Respiratory infection	2.05 (0.93–4.53)	0.074
Sepsis/Septic Shock	0.39 (0.04–2.69)	0.306
Antibiotics > 48 h	5.60 (3.92–7.98)	< 0.001
Carbapenem	1.33 (0.93–1.89)	0.108
Quinolones	1.21 (0.85–1.70)	0.297
^2^IBL	3.75 (2.83–4.97)	< 0.001
Cephalosporin	1.87 (1.38–2.53)	< 0.001
Amynoglycosides	3.78 (2.67–5.37)	< 0.001
Linezolid	3.02 (1.21–4.17)	< 0.001
^3^SDD	0.45 (0.05–3.64)	0.454

^1^COPD: Chronic obstructive pulmonary disease; ^2^BLI: b-lactamase inhibitors; ^3^SDD: Selective digestive decontamination

The univariate analysis showed that a stay longer than 7 days, the origin of the patient, an APACHE-II score exceeding 20, respiratory infection as the cause of admission, use of parenteral nutrition, and renal replacement techniques implemented along with the administration of any antibiotic for more than 48 h —especially linezolid, penicillins/b-lactamase inhibitors (BLIs), cephalosporins, carbapenems, or aminoglycosides— were associated with colonisation by strains of MR *K. pneumoniae* (Table 2). In the multivariate analysis (Table 3), hospitalisation for longer than 7 days together with respiratory infection, and administration of any antibiotic —particularly penicillins/BLIs and aminoglycosides— was associated with increased MR *K. pneumoniae* colonisation rates.

**Table 3:** Results from the multivariate analysis

	OR (IC95%)	P
Length of stay > 7 days	3.76 (1.89–5.00)	< 0.001
APACHE II > 20	1.14 (0.84–1.56)	0.400
Respiratory infection	1.75 (1.27–2.42)	0.002
Origin	1.23 (0.89–1.63)	0.327
Renal replacement therapy	1.23 (0.80–1.90)	0.136
Parenteral Nutrition	1.32 (0.96–1.80)	0.087
Antibiotics > 48 h	2.86 (1.83–4.48)	< 0.001
^1^IBL^1^BLI	1.89 (1.68–8.33)	< 0.001
Cephalosporin	1.85 (1.33–2.69)	0.214
Aminoglycosides Aminoglucósidos	2.04 (1.35–2.10)	0.001
Linezolid	1.38 (0.96–1.98)	0.098
Carbapenem	0.79 (0.53–1.16)	0.230

^1^BLI: b-lactamases inhibitors

**Fig. 2:**
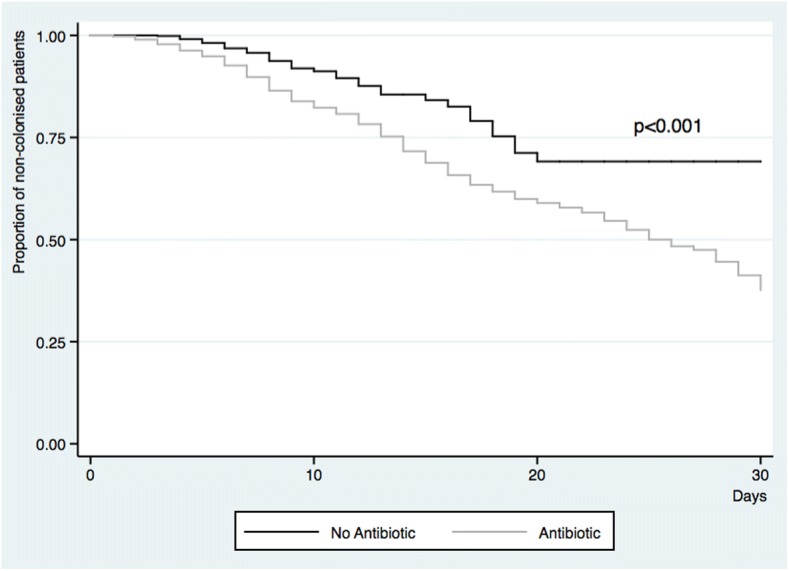
Days until *K. pneumoniae* colonisation of the patients included based on the use of antimicrobials

The effect of antimicrobial consumption on the speed with which patients were colonised in the unit is shown in Fig. 2. Patients who received antibiotics for more than 48 h were colonised earlier than patients who did not receive this treatment [hazard ratio: 2.16 (95% CI: 1.55–3.03)].

### Association between antimicrobial consumption and the colonisation rate in the unit

The ARIMA model found a significant association between the monthly colonisation rate for MR *K. pneumoniae* and the consumption of cephalosporins and carbapenems in the previous month (Table 4). No association between the overall consumption of antibiotics in the unit and the colonisation rate was found.

**Table 4:** Relationship between antibiotic use (Daily Defined Dose) and colonization

	% Colonization during the same month		% Colonization during the next month	
	Coefficient	*p* value	Coefficient	*p* value
Carbapenem	0.720	0.220	0.380	0.043*
Quinolones	−0.079	0.688	−0.086	0.669
^1^BLI	−0.048	0.856	0.065	0.787
Cephalosporin	−0.665	0.164	0.616	0.013*
AminoglycosidesAmynoglycosides	0.392	0.472	0.833	0.226
Linezolid	0.569	0.075	0.287	0.434
Total	−0.055	0.163	0.024	0.557

^1^IBL^1^BLI: b-lactamases Inhibitors

*​Statistically significant

## Discussion

The results of our work show that, in an endemic situation, the administration of antimicrobials, as well as the antibiotic pressure of cephalosporins and carbapenems, is related to increased colonisation by MR *K. pneumoniae* strains.

The expansion of MR bacteria is now one of the main concerns for critical patient units. Different authors have analysed the effect of antimicrobial consumption on colonisation by MR strains in these units, and have found a clear association between the individual consumption of antipseudomonal drugs and colonisation or infection by MR *P. aeruginosa* [17, 18] or *A. baumannii* strains [9, 19]. However, even though this species is expanding, the effect of administering antimicrobials on the rate of *K. pneumoniae* colonisation in these units has been little studied [20, 21]. Unlike previous studies [18–20], we included both rectal and respiratory samples which increased the probability of identifying patients colonised by these strains.

The results we obtained show that the length of stay in the ICU is a factor that determines colonisation by MR strains of *K. pneumoniae*, whose colonisation prevalence exceeded 50% in patients who remained in the unit for more than three weeks. However, we have also demonstrated that patients who received antibiotic treatments were colonised earlier, showing that, although the unit hospitalisation length inevitably conditions the risk of colonisation, the consumption of antibiotics is a key factor in accelerating it. Changes in the microbiota observed in critically ill patients [22, 23] could be favored by the use of antibiotics, which would bring on the selection and persistence of the most resistant strains. It should be noted that certain patients remain without being colonized despite having been admitted to the unit for several weeks and despite having received different antibiotic treatments.. It has been postulated that there could be factors specific to the flora of the host, which are currently not understood, that might act as protectors against exogenous strains [24].

Among the antimicrobials studied, use of the penicillin/BLI and aminoglycosides combination has been associated with a significant colonisation risk. The capacity of penicillins/BLIs to select MR strains is well known [25, 26]. However, data referring to the use of aminoglycosides are more controversial and disparate results have so far been reported in the literature [17, 18, 25]. Carbapenems did not appear to have any significant effect on individual patient exposure or colonisation by MR strains. This phenomenon could be explained in part because in our unit, this antibiotic is usually used in patients who are already colonised by MR strains, and who completed their follow-up in our study.

With respect to the pressure on antimicrobial consumption, very few studies have evaluated the overall effect of the use of antibiotics and colonisation by MR strains in ICU units [18]. In the present study, we found an association between the global consumption of carbapenems and cephalosporins, and colonisation in the month following the intervention. The effect produced by this group of antibiotics and their capacity to select for MR bacteria is known, having been related to a selective pressure on the microbiota [10, 26]. However, it should be noted that no association was found between the overall consumption of quinolones or BLIs and the number of colonised patients, even though these are also frequently cited in the literature as factors related to the selection of MR strains [9, 18, 27], and despite the fact we found an individual effect in the case of BLI. This current analysis shows that, although the consumption of antibiotics seems to lead to higher overall colonisation, analysis of the association between the antimicrobial-use pressure and the development of resistance is complex. It should be pointed out that the evaluation of the impact of antibiotics on in critically ill patients microbiota is highly complex because of the heterogeneity of dosing regimens, the co-administration of other antibiotics and other drugs that may also impact on its diversity. Therefore, to reduce the colonisation pressure, in addition to minimising exposure to antibiotics, it is essential to apply measures that have been shown to reduce colonisation from other patients or from colonised environmental surfaces; these measures include handwashing surveillance programs, adequate cleaning of patients, and the use of diverse strategies to minimise surface contamination [28, 29].

Among the limitations of the study is, first of all, its unicentric nature. The analysed data come from a medical unit with specific epidemiological characteristics, which may not be applicable to other units with other types of MR strains. This study has been focused on MR *K. pneumoniae*. Most of patients that have other MR bacteria, such *MRSA, E. cloacae, P. aeruginosa* o *A. baumannii* had been admitted to other units prior to their entry into the ICU, with a high suspicion that they could have been colonized in them. However, *K. pneumoniae* is an endemic bacteria of the ICU within our hospital, so we could study the effect of antibiotic use on the incidence of *K pneumoniae* colonisation better than with the set of MR strains, with a lesser influence of external factors to those of the ICU.

We have presented an ICU we a high proportion of carbapenem-produced *K. pneumoniae* colonisation with a relatively low consumption of carbapenems, which reflects the endemic situation of the unit. It should be noted that, in an endemic situation, most patients are colonised through cross-transmission via several different materials and from the health professionals themselves [30]. This is why most patients may be initially colonised on a surface of their body before later presenting colonisation of their digestive tract, from where the surveillance samples are obtained. The effect of antibiotic consumption on this type of transmission is uncertain. However, the loss of the bacterial flora because of the use of antimicrobials could facilitate this colonisation phenomenon.

Although surgical intervention has been associated with higher colonisation by MR bacteria [31], we did not include this variable as a colonisation risk factor in our study because our ICU does not see surgical patients. Similarly, we did not study the impact of the insertion of central venous catheters on colonisation, even though other authors have demonstrated that this is a risk factor for infection by MR strains [12, 25], because most of the patients admitted to our unit have this type of line. On the other hand, some patients were evaluated during their stay in our unit but not subsequently followed-up in the hospitalisation rooms. This means that the true number of colonised patients may have been underestimated. In addition, during this period, even though our ICU had agreed-upon protocols that were well-known by the unit’s staff, we were unable to rigorously analyse compliance with the various strategies in place to help prevent patient colonisation. This fact, together with the issue that the different measures were carried out at different times during the follow-up period, has prevented the effect of these interventions from being included in the analysis.

## Conclusion

Given the association between colonisation and infection, and the increase in morbidity and mortality associated with infection with MR strains [6, 13], further strategies to minimise colonisation by these strains must be implemented. In this context, minimising patient exposure to antibiotics is a fundamental factor in reducing this type of colonisation. In summary, the individual and overall consumption of antibiotics determines the colonisation of patients by MR *K. pneumoniae*. Therefore, the implementation of strategies that reduce exposure are required to reduce colonisation by these types of MR strains.

## Abbreviations

DDD: Daily defined dose
ECDC: European Centre for Disease Prevention and Control
ESBL: Extended-spectrum b-lactamases
ICU: Intensive care unit
MIC: Minimum inhibitory concentration
MR: Multiresistant
MRSA: Methicillin-resistant *S. aureus*
